# Bacterial cell fate under stress: lipid remodeling and antimicrobial peptide attack

**DOI:** 10.1038/s44259-026-00195-7

**Published:** 2026-04-02

**Authors:** Djenana Vejzovic, Theresa Schwaiger, Altea Topciu, Lukas Petrowitsch, Ajla Arnautovic, Nermina Malanovic

**Affiliations:** 1 Institute of Molecular Biosciences, University of Graz, Graz, Austria; 2 Field of Excellence BioHealth, University of Graz, Graz, Austria; 3 BioTechMed-Graz, Graz, Austria

**Keywords:** Biochemistry, Biotechnology, Drug discovery, Microbiology

## Abstract

Dynamic changes in lipid membrane composition are a common response to stress, often involving shifts in key lipid molecules. Phosphatidic acid (PA), a central precursor in lipid biosynthesis, accumulates when anionic phospholipid synthesis is blocked—lipids that are typically primary targets of membrane-active antimicrobial peptides (AMPs). This raises the question of how cationic AMPs adapt to such lipid remodeling, which is especially relevant given their promise as novel therapeutics against escalating antimicrobial resistance. Their killing mechanism is often unclear. To identify ongoing processes clearly linked to bacterial cell death, six assays targeting membrane integrity and cell viability were performed alongside bactericidal measurements. These assays were conducted on *Escherichia coli* and a mutant depleted of anionic phospholipids, treated with the cationic peptides melittin and LL-37. Correlation of assays generated characteristic antimicrobial profiles, providing insight into the peptides’ mechanisms. LL-37 acted independently of membrane composition, while melittin showed increased activity in the absence of anionic phospholipids. This study confirmed specific interactions with PA, but their action suggests targets beyond the membrane, as bacteria remained viable during membrane disruption but failed to form colonies. Overall, these findings indicate that both peptides can effectively handle lipid remodeling and uncover processes driving bacterial cell death.

## Introduction

To survive external challenges, whether from therapeutic drugs or changes in environmental conditions, bacteria have evolved a variety of adaptive strategies^1–3^. A key aspect of this adaptation is the protection of their cell envelope, which enables them to withstand hostile conditions. Remodeling of the membrane is therefore closely linked to evolution of antimicrobial resistance phenotypes^4^, a process not only governed by the genetic regulation but also by dynamic changes in the membrane lipid composition^5^. These changes can be triggered by environmental stresses such as temperature fluctuations or shifts in ionic strength, enabling bacteria to maintain optimal membrane fluidity. Adaptation involves increased incorporation of unsaturated fatty acyl chains, branched-chain fatty acids, or phospholipids of different headgroups-phospholipids inducing high or low lipid packing^5,6^. Membrane reorganization, along with disruption of lipid homeostasis that regulates membrane properties, is strongly associated with processes of regulated cell death in eukaryotes, from apoptosis to lipid peroxidation^7^. However, the regulated mechanisms of bacterial cell death remain poorly understood. Although bacteria do not undergo apoptosis as seen in eukaryotes, their death processes are often associated with environmental stress or damage. For example, jellyfish tolerate deep-sea pressure by incorporating plasmalogens, lipids with high negative curvature, into their membranes, preserving flexibility under extreme conditions^8^. The introduction of plasmalogen synthesis in *Escherichia coli* enhanced their tolerance to high pressure, whereas strains engineered with rigid, cylindrical lipids exhibited mortality under similar conditions due to excessive membrane packing. Under high pressure, increased lipid packing reduces membrane fluidity and alters lipid shape, potentially disrupting membrane protein function and impairing essential processes like membrane fusion and fission. Altogether, regardless of the type of environmental pressure, osmotic, hydropressure or mechanical, that deforms or alters the membrane, bacteria must modify their membrane properties to regulate ion flux and adjust cytoplasmic solute concentrations^9^. These adaptations are essential for maintaining turgor pressure, membrane integrity, and overall cellular homeostasis, enabling bacteria to preserve function and survive under varying stress conditions. Under selective pressure, particularly mechanical-like stresses such as exposure to antibiotics/antimicrobial peptides, bacteria can alter their lipid composition, leading to changes in membrane thickness, density, and surface charge^10,11^. A well-known example of lipid-based adaptation is polymyxin resistance^12^, or in the case of *Staphylococcus aureus*^11^, where bacteria modify their membrane lipids, such as through the addition of arabinose, cationic amino acids or alanine, to reduce the negative surface charge and limit antibiotic binding. In more extreme cases, antibiotic treatment can lead to complete structural deformation, as seen when bacteria lose their cell wall and transition into cell wall-deficient forms such as L-form bacteria or mycoplasma-like states. These forms exhibit dramatically altered membrane compositions, often incorporating atypical lipids for bacteria, such as phosphatidylinositol or neutral phospholipids, which are more characteristic of eukaryotic membranes^10^.

Targeting the membrane composition of cells is a well-established strategy in combating disease^7^. One effective approach to modifying bacterial membrane properties and induce cell death is through the use of membrane-active agents. They offer a promising potential in a fight against various infections caused by multi-drug resistant pathogens, a major global health threat^13^. This is especially relevant in the current era of the silent pandemic era, where life-threatening secondary fungal^14^ or bacterial^15^ infections are frequently associated with Covid-19 [2] or cancer^16–19^. One such membrane-active compound is octenidine, an antiseptic with broad antimicrobial activity and no specific molecular target, which has been used in clinical settings for over three decades without the emergence of clinically relevant resistance^20–23^. Octenidine strongly disrupts lipid packing within the acyl chain region of the membrane^21^, *Pseudomonas aeruginosa* resistant colonies survived octenidine treatment due to lipid remodeling mediated by mutations in *phosphatidylserine synthase* (*pssA*) and *phosphatidylglycerolphosphate synthase* (*pgsA*)^24^. However, the reason for impaired octenidine efficacy in these cases remains unclear, as its activity is largely independent of lipid headgroup composition^20,22^ and mutants lacking the same lipid species in genetically modified bacteria did not show reduced susceptibility^20,22^, and no octenidine resistance has been identified in clinically relevant pathogens^25^. Another class of membrane-active compounds with increasing therapeutic potential against bacterial infections are AMPs, whose use has been studied more extensively over the past decade^10,11^. Despite limitations that hinder their widespread clinical application, such as stability, toxicity, or the translation of the killing potency from in vitro to in vivo, AMPs offer advantages like rapid action and a lower likelihood of resistance development compared to conventional antibiotics^26–28^. This is largely due to their broad-spectrum activity against various bacterial pathogens^28^, primarily through targeting the fundamental barrier function of the cell membrane via disruption—although the composition of the cytoplasmic lipid membrane can vary across bacterial populations^10,11,29^. However, as in many other cases, the full extent of the effects that AMPs exert on microorganisms remains poorly understood. At the cellular level, AMPs typically affect membrane integrity by depolarizing the membrane, altering fluidity, increasing permeability, and disrupting lipid packing^30–32^. Yet, it is often unclear whether direct membrane disruption is the immediate cause of cell death, or if death results from downstream effects following altered membrane properties like protein delocalization and intracellular leakage^32,33^. In general, the mode of action (MOA) of AMPs, respectively, is strongly determined by their chemical and physical properties like hydrophobicity, charge, structural flexibility, hydrogen bonding capacity and most importantly their amphipathic character^10,11,29^. In turn, variations in membrane lipid composition, such as differences in charge, fluidity, and lipid packing, can influence bacterial susceptibility to specific AMPs by affecting peptide binding, insertion, or membrane-disruptive efficiency.

In this context and given the significantly low number of AMPs currently in the clinical pipeline^34^ several key questions remain unanswered: (i) which specific mechanisms of membrane disruption occur upon AMP attack; (ii) which mechanisms directly contribute to or ultimately result in bacterial cell death; (iii) how AMPs interact with the cytoplasmic membrane in the absence of major anionic phospholipids; and (iv) how AMPs maintain their disruptive activity when bacteria undergo lipid remodeling?

In order to understand mechanism underlying cell death by AMP we selected the two most studied membrane-active peptides, honey bee venom melittin^35^ and human cathelicidine LL-37^36^ and investigated their activities in a very similar fashion thereby using specific markers for cell death and markers detecting changes on the membrane. LL-37 and melittin also show major differences in their interactions with membranes and modes of action, ranging from pore formation to a carpet-like mechanism^37–42^. These differences appear to be driven by their distinct amphipathic α-helical structures and the distribution of hydrophobic and charged residues. Melittin has a predominantly hydrophobic N-terminal region and a hydrophilic C-terminal region^43^, whereas in LL-37, cationic and hydrophobic residues are segregated along opposite sides of the helix^44^. Their orientation and insertion into the membrane are highly dependent on lipid composition, which further influences their mechanism of action^40,45–48^. For this purpose, experiments were performed on *E. coli* and mutant defective in production of anionic phospholipids, phosphatidylglycerol (PG) and cardiolipin (CL) which are supposed to be the major interaction partners of AMPs on bacterial membranes. Here we show, which changes affecting membrane integrity ultimately correlate with markers of cell viability and result in cell death of bacteria.

## Results

### Melittin and LL-37 interact with phosphatidic acid and associate on membranes similarly to phosphatidylglycerol/cardiolipin rich membranes

**Fig. 1:**
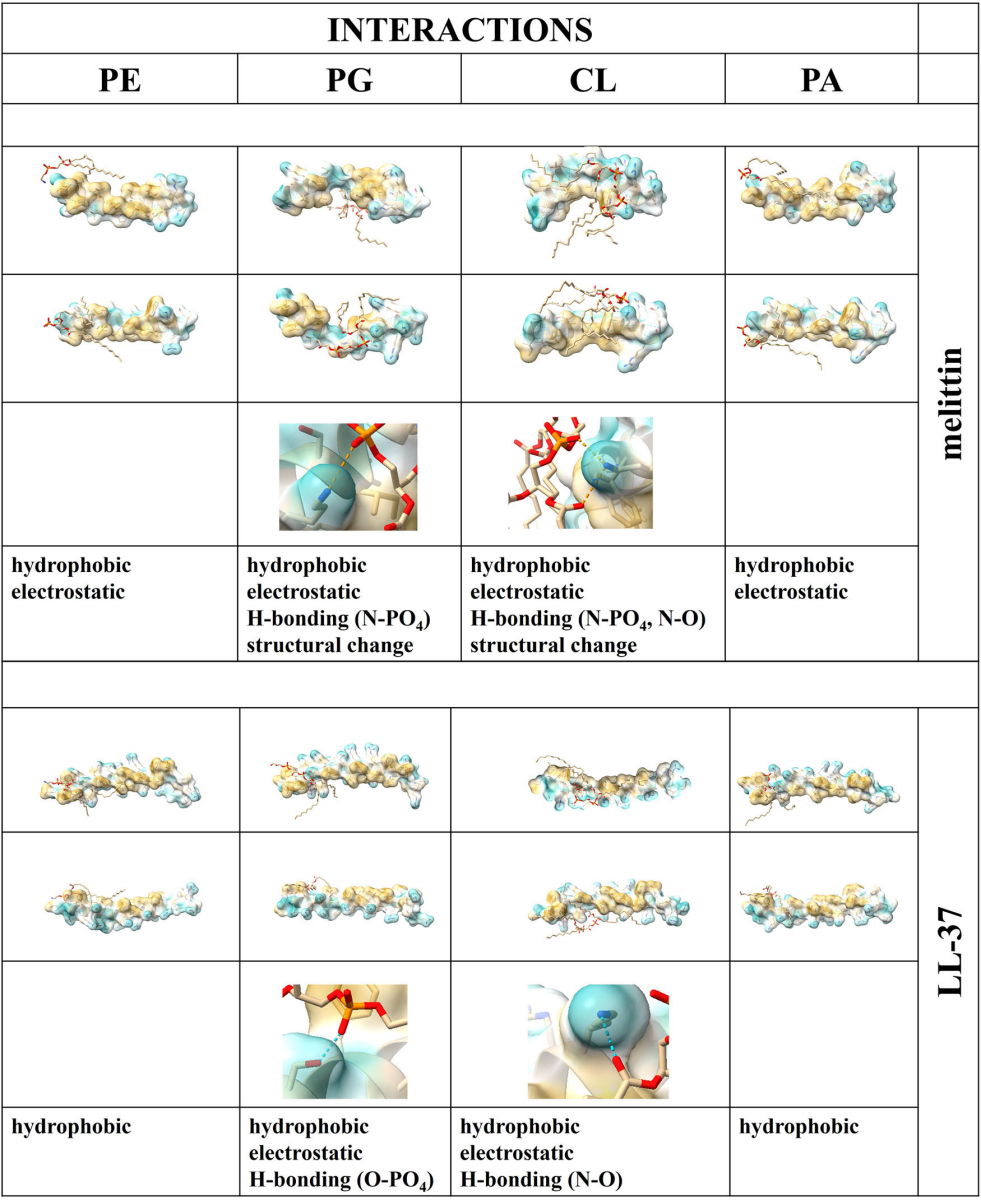
AlphaFold 3 predicts hydrophobic interactions between melittin and LL-37 with both anionic and zwitterionic bacterial phospholipids. Prediction of all of these models showed pTM scores between 0.71 and 0.77 and ipTM scores between 0.68 and 0.75.

**Fig. 2:**
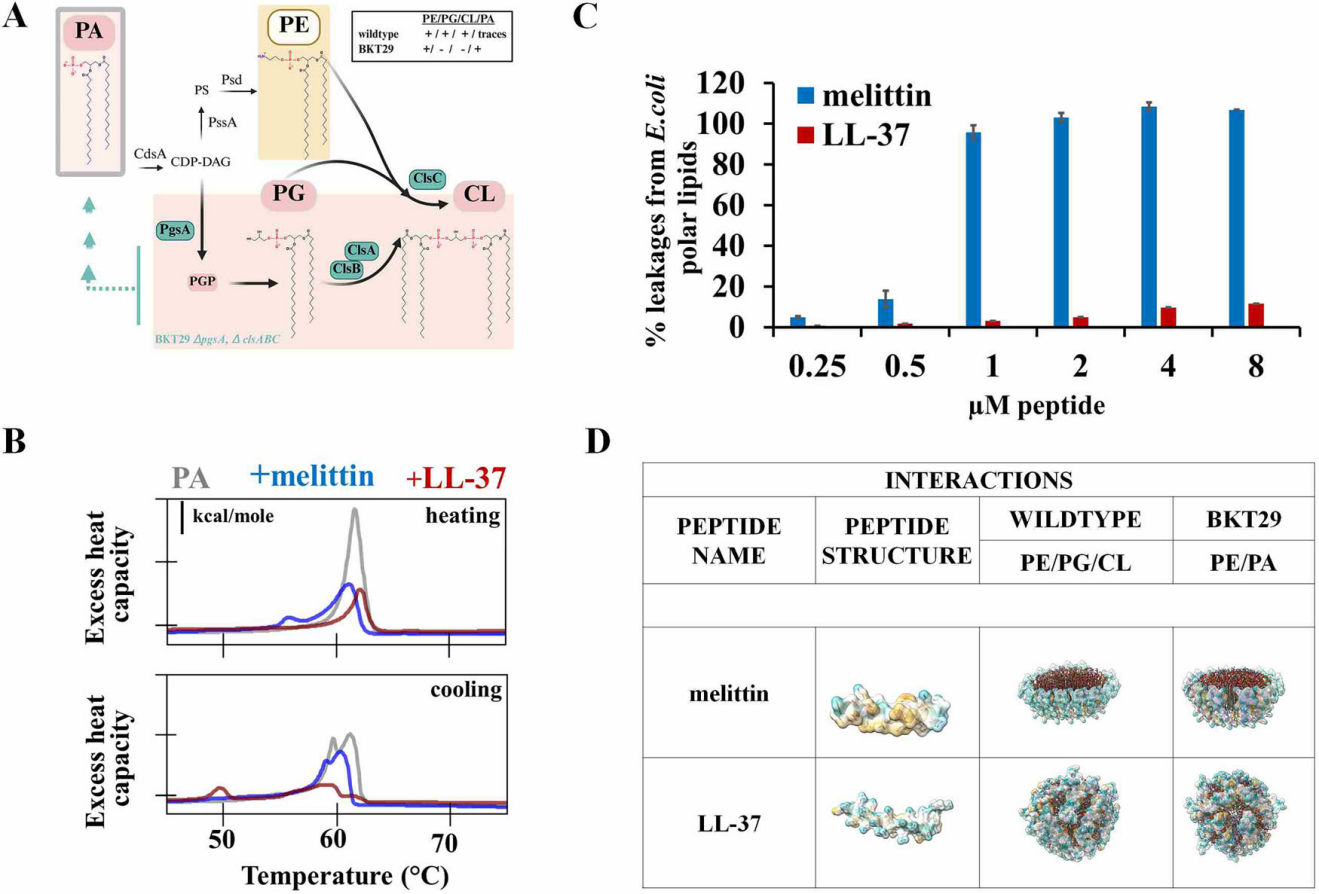
Melittin and LL-37 interact with remodeled membranes similarly to wildtype. **A** Phospholipid metabolism in *Escherichia coli* (**B**) Heating and cooling scans of PA phase transition exposed to peptides. The experiments were conducted on large multilamellar vesicles comprising PA phospholipid, with and without LL-37 and melittin, at a 25:1 lipid-to-peptide molar ratio. Representative molar heat capacity profiles for PA (grey), PA/LL-37(red) and PA/melittin (blue) mixtures after buffer subtraction, as measured by differential scanning calorimetry in duplicates. **C** Leakage of ANTS/DPX incorporated dye from 50 µM LUVs generated from *E. coli* lipid polar extracts. Experiments are mean results of two independent experiments. **D** AlphaFold 3 prediction of the interaction of LL-37 and melittin with different bacterial membranes, mimicking wildtype and BKT29 membrane lipid composition. Prediction analysis was performed with 14 peptide molecules and 100 lipid molecules consisting of PE, DPPG, DSPG, and CL (67:13:12:10) for *E. coli* wildtype membranes and PE and PA in a ratio of 3:1 for the membrane of the BKT29 strain. Prediction scores are on the low end (0.56/0.59 for LL37 and 0.88 for both membranes for melittin) as lipids are mostly flexible and randomly distributed.

Numerous studies demonstrate non-selective interaction of melittin and LL-37 with phospholipids^35,37–43,45–51^, highlighting the impact of complex interplay of headgroup charge, chain packing density, hydrocarbon chain length, hydrogen bonding capacity, and lipid molecular shape. However, for none of the AMPs studied has the mode of action remained as unclear as for these two, particularly in terms of how their activity translates to real bacterial membranes. To further get an overview of the interaction between peptide and lipid at molecular level we predicted the fold of the peptides together with each lipid by using a structure-prediction tool AlphaFold 3 (Fig. 1). In Gram-negative bacteria, phospholipid metabolism is relatively simple, with phosphatidic acid (PA) serving as a precursor for the synthesis of the major cytoplasmic membrane lipids: phosphatidylethanolamine (PE), phosphatidylglycerol (PG), and cardiolipin (CL) (Fig. 2A)^52,53^. We have classified the interaction of peptides with earlier mentioned lipids according to the kind of interaction we see within the predicted models from pure hydrophobic interaction over electrostatic, in case head groups would be placed near side chains of opposite charge, to hydrogen bonds. Interestingly, we observed a structural change of melittin when the structure was predicted in presence of PG and CL leading to a curvature around the lipid molecule. Both peptides showed preferential binding to PG and CL, suggesting these anionic lipids play a central role in AMP recruitment and alignment. Although PA is an anionic lipid, its interactions with both peptides appear to be primarily driven by hydrophobic forces. To further evaluate their membrane-disruptive potential, we investigated the capacity of the peptides to permeabilize membranes composed of *Escherichia coli* polar lipid extracts (Fig. 2B). Interestingly, melittin induced complete release of ANTS/DPX from these membranes at a concentration as low as 1 µM, while LL-37 failed to elicit significant leakage, even at concentrations up to eightfold higher than those required for melittin. Although LL-37 has previously been reported to permeabilize membranes composed of *E. coli* lipids at the same lipid-to-peptide ratio used here^49,54^, our data indicate that LL-37 is markedly less effective than melittin in compromising membranes derived from *E. coli* polar lipid extracts. This discrepancy likely arises because the absolute lipid and peptide concentrations may vary between assays, and factors such as incomplete binding, peptide distribution or peptide aggregation can impact the effective peptide concentration per vesicle, influencing membrane disruption. Nevertheless, our data align with previous reports showing a lack of LL-37 interaction with PE^46^. Because PE is a major component of *E. coli* polar lipids, this likely explains its limited membrane disruption. As anionic phospholipids are generally reported to be the essential interacting partner of membrane-active compounds, the BKT29 mutant strain depleted in genes essential for PG and CL synthesis, was chosen to better understand the importance of charged phospholipids in the bacterial membrane in respect to the antimicrobial activity of the melittin and LL-37. Although bacterial lipid composition is diverse, *E. coli* is a well-established model for Gram-negative organisms because its cytoplasmic membrane composition is broadly representative and, importantly, permits experimental manipulation of anionic phospholipids, which is not feasible in most Gram-positive bacteria where these lipids are essential for viability^11^. In our previous study we confirmed the absence of PG and CL in BKT29^53^. However, the loss of membrane anionic charge in this mutant was partially compensated by the accumulation of phosphatic acid (PA), which is normally present only in trace amounts ( ~ 1%) in the wildtype. Because interactions with the PA are less explored, we then monitored the effect of the LL-37 and melittin on the thermotropic behavior of the PA phospholipid bilayer by using differential scanning calorimetry (DSC). The DSC curve for pure PA (Fig. 2B in grey) was characterized by a single endothermic peak at 61.8 ± 0.1 °C and an exothermic peak at 61.3 ± 0.1 °C, representing the main phase transition of the lipid bilayer: the transition from the gel phase (L_β´_) to the liquid crystal phase (Lα) and vice versa. During the cooling phase, there was no single defined peak for PA, but rather a split one, likely due to a slower or more heterogeneous phase transition. In the presence of melittin (Fig. 2B in blue), phase separation into two distinct peaks was observed during both heating and cooling scans. The dominant endothermic peak shifted to a lower temperature, reducing the main transition temperature by at least 0.6 °C during heating and 1 °C during cooling. Additionally, the overall enthalpy decreased (from 8.4 ± 0.1 to 6.6 ± 0.2 kcal/mole), indicating that melittin inserts into the PA bilayer and likely disrupts its organization. These effects are probably driven by electrostatic interactions between the negatively charged headgroup of PA and the five positively charged residues of melittin, facilitating its initial anchoring to the bilayer. However, given the observed reduction in both transition temperature and enthalpy – signs of deeper insertion and structural disruption – hydrophobic interactions between melittin and PA are also likely to contribute. In the case of LL-37 (Figure 2B in red), a slight increase in the main transition temperature by ( + 0.5 °C) and a decrease in transition enthalpy to 2.9 ± 0.2 kcal/mole were observed during heating scans. This was accompanied by phase separation and further reductions in transition enthalpy and temperature during cooling scans. Compared to melittin, the effects induced by LL-37 appear more pronounced. Electrostatic interactions may also be stronger in this case, as LL-37 contains 11 positively charged residues. These results confirm the capability of the peptides to interact with PA in a manner comparable to their interactions with other anionic phospholipids^45,46,50,55^. To further characterize the thermodynamic contributions to peptide–PA interactions, we estimated the apparent enthalpy and entropy changes from the calorimetric data. For melittin, the transition entropy decreased from 0.03 to 0.02 kcal/ mole/·K, while for LL-37 it dropped to 0.01 kcal/mol/·K. The apparent Van’t Hoff enthalpy, analyzed using a Gaussian fitting model, decreased from 7.8 ± 0.6 to 5.4 ± 0.3 kcal/mole for melittin and to 2.6 ± 0.2 kcal/mole for LL-37, with the reduced Van’t Hoff ratio (CU = ΔHvH / ΔHcal) declining from 0.92 to 0.81 for melittin and reaching 0.90 for LL-37. These changes indicate that peptide binding disrupts cooperative lipid domains while slightly stabilizing residual gel-phase regions, consistent with a surface-associated, electrostatically guided interaction that is complemented by hydrophobic insertion, as suggested by the shifts in transition temperatures and enthalpies. Furthermore, consistent with AlphaFold 3 results (Fig. 1), these findings suggest that hydrophobic interactions are the preferred mode of peptide binding to PA. To gain structural insight into how such membrane compositional changes might influence AMP behavior, we again employed AlphaFold 3^56^, that was also recently extended to accommodate multimeric protein–ligand interactions. Prediction analysis was performed with peptides and membranes resembling the phospholipid composition of the wildtype and BKT29. Consistent with their known antimicrobial actions^37,38,57^, for LL-37 a carpet like mechanism is predicted with the peptides aligning alongside a lipid monolayer while melittin in contrast exhibit vertical orientation with peripheral anchoring at the monolayer edge, without intercalation between lipid molecules (Fig. 2D). One might argue that this behavior suggests the potential for pore formation; however, no channel-like structures were observed, as have been recently described in simulation models^51^. This edge-associated vertical binding may reflect a specific affinity for defect-prone or energetically exposed boundary regions, rather than a mechanism involving deep insertion, full translocation, or pore formation. No significant difference in the mode of binding could be observed between LL-37 binding to the two different bacterial model membranes while melittin, even though binding generally in the same way, seems to form a denser and more compact binding when interacting with the PA-rich BKT29 membrane compared to the wildtype membrane. Nevertheless, while AlphaFold 3 provides qualitative insights into peptide–lipid interactions, these predictions are intended to complement experimental data rather than serve as definitive models, and more detailed simulation approaches such as molecular dynamics would be required for a full mechanistic understanding.

**Fig. 3:**
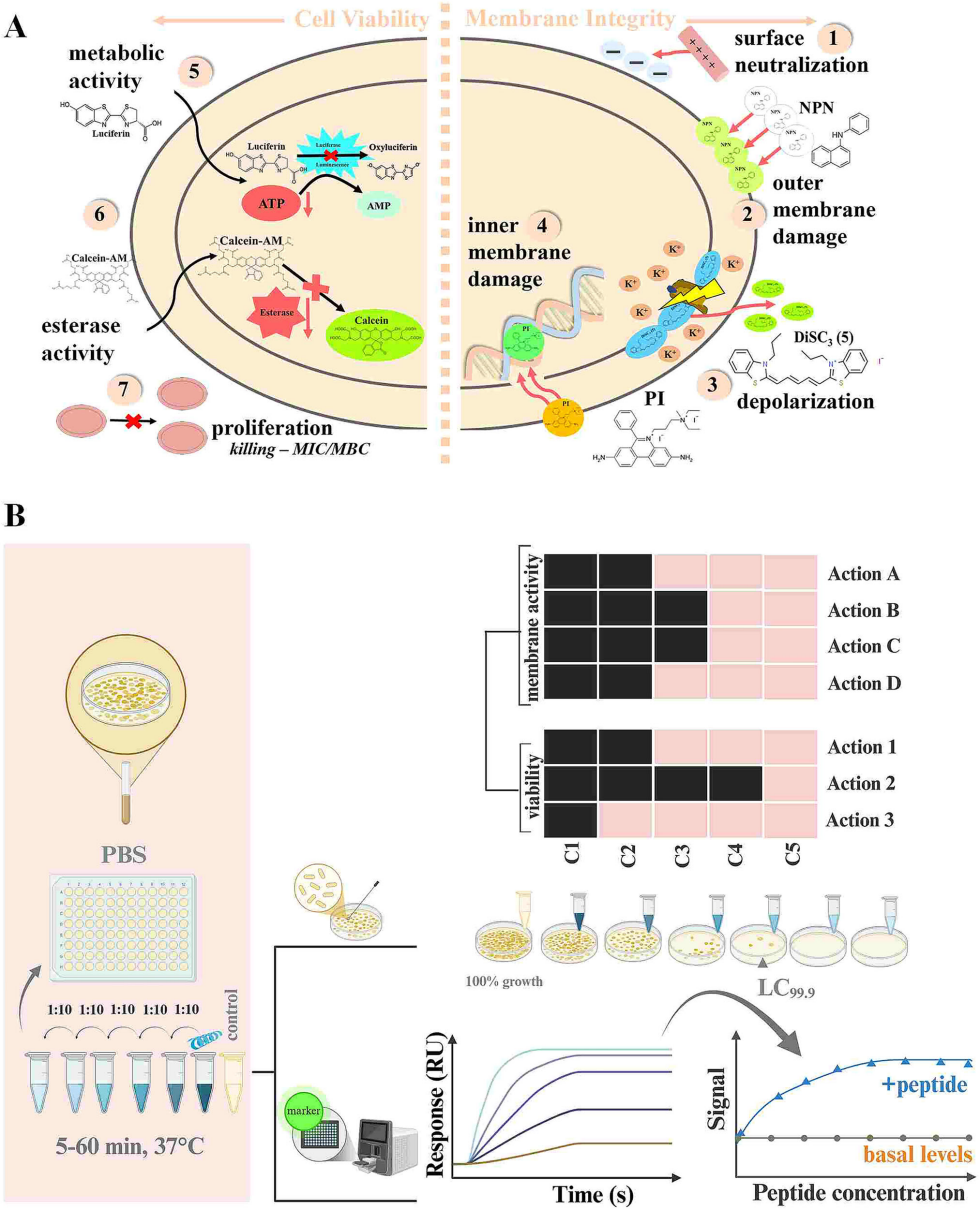
Overview and schematic theoretical background of performed methods. **A** Actions of membrane-active compounds are indicated by red arrows 1. Assessment of surface charge is performed through zeta potential measurements, as cationic membrane active compounds neutralize the negatively charged bacterial membrane 2. If outer membrane damage occurs caused by AMPs the non-polar dye NPN can get into the hydrophobic environment, where fluorescence intensifies 3. The self-quenching DiSC_3_(5) embeds itself into the inner membrane, where it is released and dequenched upon changes in membrane potential by treatment with AMPs 4. The membrane impermeable PI can only penetrate compromised inner membranes, where it fluoresces upon binding to DNA giving information about membrane permeabilization. 5. ATP levels as indicators of metabolism are measured by the ATP-dependent oxidation of luciferin to oxyluciferin 6. Intracellular esterase activity is reported to be a marker of viable bacteria, which can be assessed based on the esterase-catalyzed conversion of calcein-am to calcein. 7. The Minimum Inhibitory or Bactericidal concentration (MIC/MBC) are parameters measured by determination of the optical density at 600 nm of bacterial suspensions or by colony counting. **B** Schematic workflow and analysis of the results.

While model membranes offer valuable insights into molecular interactions, we further focus on interactions with bacteria to explore these processes at the cellular level in a physiologically relevant context. To this end, such interactions typically proceed through a series of sequential steps: (i) binding to the bacterial surface, (ii) permeabilization of the outer membrane, (iii) disruption of the inner (cytoplasmic) membrane, (iv) leakage of intracellular contents, and (v) ultimately, cell death. To evaluate these processes and assess the effects of the tested compounds on both membrane integrity and bacterial viability, we performed a series of assays, which are summarized in the overview presented in Fig. 3. Also, the theoretical background of the assay is demonstrated in a schematic way for every assay, including the used fluorescent dyes in the membrane integrity assays and the substrates/enzymes for the reactions regarding cell viability. Evaluating the assays and putting them into relation to the killing potential of the respective compounds, should generate a characteristic antimicrobial profile for every membrane-active compound in regard to the two *E. coli* strains. Because the experiments were performed in an analogous manner, the resulting diagrams represent a snapshot of simultaneously occurring events describing actions. The assays mentioned describe effects induced by antimicrobial compounds at the bacterial surface, the inner and outer membranes as well as the influence on intracellular components giving insight into the molecular mode of action of the compound of interest. The effects of AMPs are highly dependent on peptide concentration or bacterial density—factors that contribute to variability in literature. Therefore, we strictly standardized experimental conditions in subsequent assays to ensure better comparability and more reliable interpretation of the results.

### Melittin is more potent in the absence of phosphatidylglycerol and cardiolipin

**Fig. 4:**
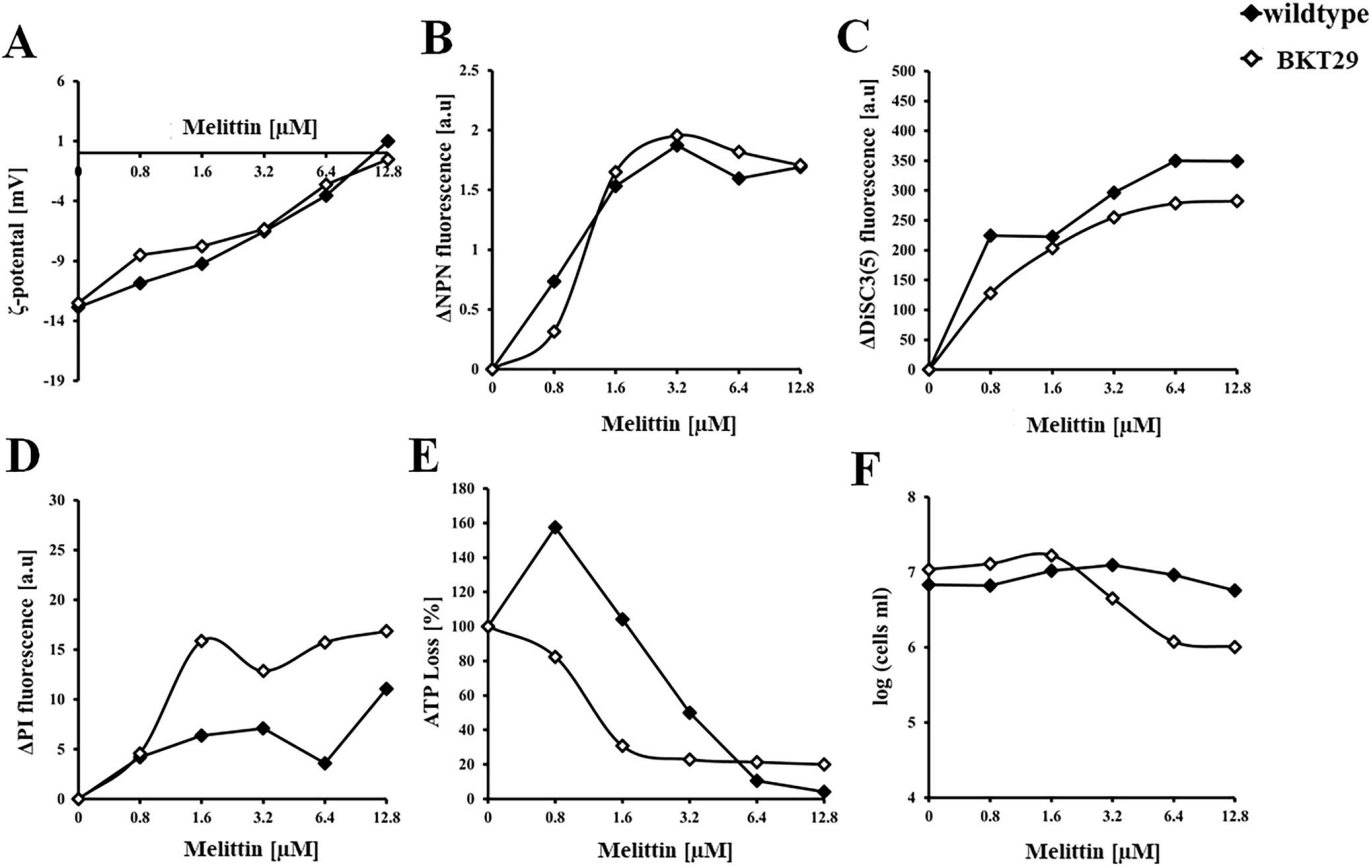
Effect of melittin on bacterial membrane and viability. Diagrams show curves recorded for *E. coli* wild type (open diamonds) and BKT29 mutant (filled diamonds) for (**A**) Zeta potential; (**B**) Outer membrane assessment; (**C**) Inner membrane potential assay; (**D**) Inner membrane permeabilization; (**E**) ATP Loss determination induced by melittin; (**F**) Viable cell measurement by quantification of intracellular esterase activity. Data are presented as medians derived from a minimum of three independent experiments.

**Fig. 5:**
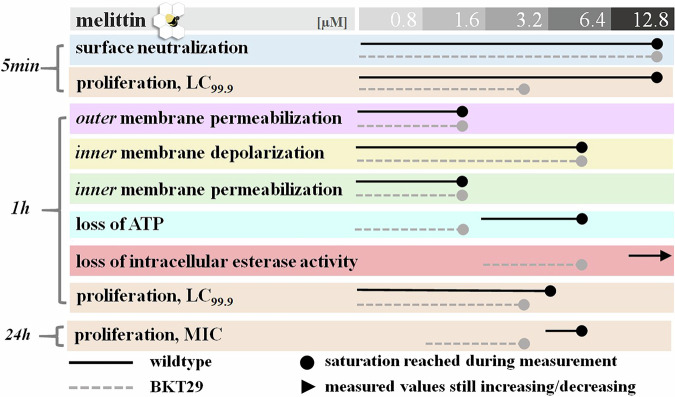
Melittin is more potent in the absence of phosphatidylglycerol and cardiolipin. Overview of ongoing activities of melittin shown as a time, concentration and lipid dependent antimicrobial profile of melittin.

To correlate membrane activity with cell viability, we first determined the bactericidal concentration under the same conditions and time frame used in the cell-based experiments. This concentration, expressed as the lethal concentration (LC₉₉.₉), was estimated under non-dividing conditions by incubating bacteria with peptides in PBS. To complement this, we also performed a standard MIC assay by measuring the optical density of bacterial suspensions over a 24 h incubation period in nutrient-rich medium. These assays revealed that melittin was markedly more effective against *E. coli* strains depleted of the anionic phospholipids. A concentration of 6.4 µM melittin is needed to inhibit the visible growth of the *E. coli* wildtype, while for inhibiting the growth of BKT29 only 1.6–3.2 µM melittin are necessary. During the initial 5-minute incubation melittin concentration of 12.8 µM eradicated 99.9% of wildtype cells, while only 3.2–6.4 µM were required for 1 h incubation. In BKT29 the LC_99.9_ concentration did not change between 5 min and 1 h and remained constant at 3.2 µM. Initial binding to the bacterial surface was assessed by measuring the zeta potential, which decreased from −13 mV in untreated bacteria to values near zero at 12.8 µM melittin (Fig. 4A). Melittin neutralized the surface charge of BKT29 in a similar manner. Interestingly, complete surface charge neutralization in wildtype occurred at the same concentration at which bacterial killing was observed (Fig. 5), highlighting that initial peptide binding to the bacterial surface is a critical step in the mode of action. In contrast, BKT29 was not completely neutralized at the LC_99.9_. Figure 3B–D shows results related to membrane permeability. In all cases, the signal from the corresponding fluorescence markers began to increase upon addition of the lowest melittin concentration. Both outer and inner membrane permeabilization assays showed saturation of the fluorescence signal at approximately 1.6 µM melittin for both strains (*p* < 0.005). The DiSC₃(5) assay, which reflects inner membrane depolarization, exhibited a similar trend (*p* < 0.005) but reached saturation only at higher concentrations ( ~ 6.4 µM). These findings suggest that the different membrane-disrupting processes occur in parallel (Fig. 5), with outer and inner membrane permeabilization preceding cell death. The continued increase in membrane depolarization beyond the bactericidal concentration indicates that significant depolarization may occur post-mortem, likely due to leakage of intracellular contents. DiSC₃(5) is a voltage-sensitive dye that responds to changes in membrane potential, particularly due to the efflux of K⁺ ions, which may increase with membrane damage^58^. A similar pattern was observed for melittin’s action on BKT29 (*p* < 0.005); however, in this case, cell death occurred at the same concentration at which membrane permeabilization was detected.

To assess metabolic activity, we measured intracellular ATP levels and esterase activity (Figure 4E, F and Fig. 5) as indicators of the cell’s energetic status. In both *E. coli* strains, ATP content decreased progressively with increasing melittin concentrations, reaching a minimum at 6.4 µM (*p* < 0.03) for the wild-type and at 1.6 µM (*p* < 0.08) for the mutant strain. Interestingly, in the wildtype strain, ATP levels remained relatively high (approximately 100%) at 1.6 µM melittin, a concentration at which membrane depolarization and permeabilization were already saturated. This suggests that membrane integrity is compromised before a substantial loss of intracellular ATP occurs. In contrast, melittin had no significant effect on esterase activity in the wildtype strain within the tested concentration range; even at the highest concentration, esterase activity remained stable during 1 h of incubation. The observation that bactericidal activity is evident while cells still retain considerable metabolic activity and energy implies that, at lethal melittin concentrations, cells may remain metabolically active but are unable to proliferate due to severe membrane damage. This indicates that melittin compromises the ability to form colonies primarily through membrane disruption, rather than by immediately halting all metabolic processes. In the absence of PG and CL, treatment with 6.4 µM melittin (*p* < 0.002) led to a drastic reduction in intracellular esterase activity, corresponding to a > 90% decrease in viable cell counts. In contrast, ATP levels remained relatively constant from 1.6 µM (*p* < 0.04) onwards but did not return to the basal levels observed in the wildtype. Interestingly, in this strain, loss of membrane integrity occurred in parallel with the decline in energy levels and bactericidal activity. This suggests that in BKT29, the absence of PG and CL compromises overall cellular fitness, making the cells more susceptible to melittin-induced killing.

In summary, in the wild-type strain, membrane impairment appears to begin simultaneously but proceeds sequentially, with depolarization and permeabilization occurring before the onset of cell death. In contrast, in the mutant strain, these events overlap more directly with cell death, suggesting a tighter coupling between membrane disruption and loss of viability. Additionally, the observation that cells retain metabolic activity even at concentrations exceeding their LC₉₉.₉ supports the conclusion that melittin exerts its bactericidal effect primarily through severe membrane damage, which impairs the ability of cells to form colonies despite residual metabolic function.

### LL-37 acts independently of phosphatidylglycerol and cardiolipin

**Fig. 6:**
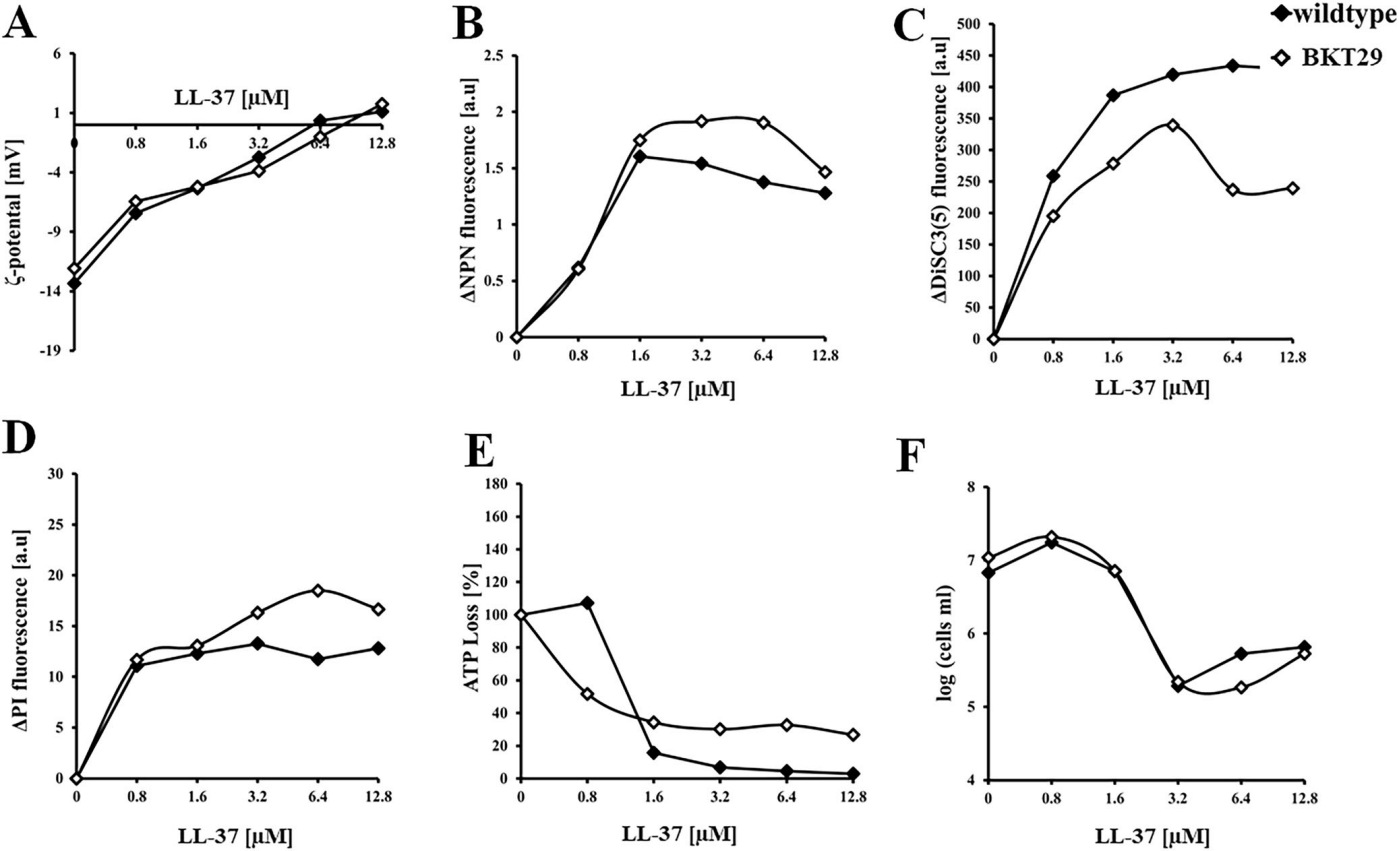
Effect of LL-37 on bacterial membrane and viability. Diagrams show curves recorded for *E. coli* wild type (open diamonds) and BKT29 mutant (filled diamonds) for (**A**) Zeta potential; (**B**) Outer membrane assessment; (**C**) Inner membrane potential assay; (**D**) Inner membrane permeabilization; (**E**) ATP Loss determination induced by LL-37; (**F**) Viable cell measurement by quantification of intracellular esterase activity. Data are presented as medians derived from a minimum of three independent experiments.

**Fig. 7:**
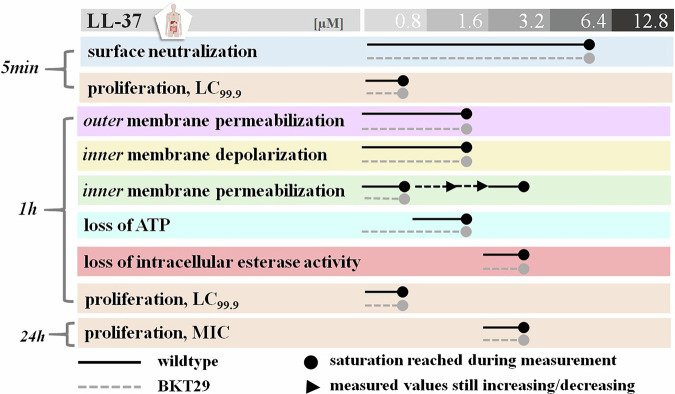
LL-37 acts independently of phosphatidylglycerol and phosphatidylcholine. Overview of ongoing activities of LL-37 shown as a time, concentration and lipid dependent antimicrobial profile of LL-37.

Like melittin, LL-37 is able to neutralize the surface of both *E. coli* strains whether anionic phospholipids are present or not. In contrast to melittin, the total neutralization of bacteria was achieved at 6.4 µM while the bactericidal effect occurred at concentrations below 1.6 µM (Figs. 6, 7). Interestingly, it could be observed that all actions involving inner or outer membranes behaved similarly as in all cases around 1.6 µM LL-37 were sufficient for signal saturation. Within the 1 h time frame all three membrane assays almost reached their signal saturation using a maximum LL-37 concentration of 1.6 µM. At a concentration of 3.2 µM (*p* < 0.005), 25% of the total inner membrane permeabilization observed in the wildtype was detected, after which the signal plateaued up to 12.8 µM, indicating that most of the propidium iodide (PI) signal had already entered the cells. Since the majority of the signal was detected by this point, it can be concluded that significant membrane disruption occurred early in the concentration range. Within the same incubation time using intracellular esterase activity as a biomarker, viable cells could however still be detected at this concentration point. In general, no significant differences in any of the six assays could be observed between the two *E. coli* strains, as LL-37 induced the same time and concentration-dependent effects with and without anionic phospholipids PG and CL present (Fig. 6) providing clear evidence for a non lipid-specific profile in regards to the two strains used. Furthermore, ATP levels only reached close to 0 values for the LL-37 treated wildtype cells as opposed to the mutant, where ATP levels remained at approximately 40% even above the lethal concentration. Of note, a clear trend in ATP loss was observed in both wildtype and mutant strains upon treatment; however, statistical significance was only reached in the mutant at concentrations around 6.4 µM (*p* < 0.03). Using intracellular esterase activity as a viability indicator, 3.2 µM LL-37 (*p* < 0.003) was required to kill both *E. coli* strains equally, while the lethal concentration (LC₉₉.₉) was reached below 0.8 µM. This supports the idea that LL-37–induced membrane damage markedly impairs bacterial proliferation, even when metabolic activity remains detectable. Since the MIC also occurred at 3.2 µM, coinciding with the complete loss of intracellular esterase activity, it can be concluded that LL-37 abolishes bacterial vitality at this concentration. Indeed, no recovery of growth was observed at concentrations up to 12.8 µM.

## Discussion

Due to rising antimicrobial resistance, developing new therapeutic strategies and antimicrobials is increasingly important. Proper dosing is critical, as suboptimal levels can promote resistance by allowing bacterial survival^59^. Initial screening of new compounds often involves assessing MIC and MBC values, which, if accurately measured, indicate antimicrobial efficacy. However, their reliability depends on correct methodology and clinical interpretation^60^. Understanding antimicrobial activity is essential before advancing compounds to clinical trials. Additionally, insights into the mechanisms of a drug can aid resistance management^28,61^. Common assays measuring cell death indicators may not directly correlate with MIC/MBC due to varying conditions, so using multiple assays can offer a more complete picture of an antimicrobial’s mode of action. This is particularly challenging for AMPs, as their cationic nature causes them to bind to plastic surfaces and components in testing media, often reducing their observed activity^26,53^. They are also highly sensitive to factors like cell density, making accurate assessment more complex^62^. As a result, we currently lack robust, translationally relevant methods for evaluating AMPs. Without this, many AMPs fail to successfully transition from preclinical research into clinical application. Another major challenge is demonstrating whether AMPs are truly superior to conventional antibiotics—specifically, whether they can effectively kill bacteria without inducing resistance and how they cope with membrane alterations when primary target molecules are absent. To address this, we applied several cell viability markers to assess bacterial cell death in response to two well-known AMPs. This approach aims to provide deeper insight into their bactericidal potential and resistance-inducing capacity, contributing to a broader understanding of their therapeutic relevance.

Taken together, our data clearly indicate that changes in lipid composition impact the antimicrobial activity of melittin. Specifically, bacterial killing by melittin is enhanced in the absence of anionic phospholipids. While melittin can breach both neutral and charged membranes, its strong interaction with anionic phospholipids in wildtype membranes appears to limit its antimicrobial efficacy. Notably, melittin’s activity increases nearly threefold in the absence of PG and CL. However, this difference appears to be substantial only during the first 5 min, as the antimicrobial activity of the wildtype increases with prolonged incubation. In the mutant lacking PG and CL, the same concentration of melittin (similar also for LL-37) induces membrane damages and initiate the metabolic rate to go down. In contrast, in the wildtype, reduction of ATP content needs higher concentrations, indicating that although the membrane is being damaged, the metabolic rate is still being kept intact. A similar trend was observed when measuring intracellular esterase activity, confirming the viability of the cells. This would indicate that the damages in the membrane at lower concentrations do not have as much of an impact on metabolic activity. A study using the same bacterial wildtype strain, *E. coli* MG1655, could provide a possible explanation for this phenomenon^40^. This study reported a melittin-induced transient permeabilization to GFP of both the outer and inner membrane of live cells. Using fluorescence microscopy, results revealed that those permeabilization sites occurred at the curved membrane surface, where the anionic PL, CL and PG, are known to accumulate^63^. These disruptions were short-lived and both membranes, inner and outer, were resealed after approximately 10 s and again repermeabilized after a few minutes. Another study revealed similar assumptions^64^. While it is plausible that melittin interacts strongly with anionic bilayers through electrostatic forces—hindering its ability to insert and adopt a transmembrane orientation due to a high kinetic barrier—the exact mechanism of its insertion and alignment to bacterial membranes has been debated for over 50 years^65^. Nonetheless, it is well established that melittin can adopt different conformations depending on membrane composition and assay conditions^64,66^. In our AlphaFold 3 prediction model, we observed a vertical orientation and peripheral anchoring of the peptide at monolayer edges, suggesting a specific affinity for defect-prone or energetically exposed boundary regions. This behavior is consistent with a boundary-stabilizing mode of action and supports the formation of transient pores that can readily reseal upon peptide disengagement, rather than stable membrane penetration. Resealing of membranes, as observed for damage induced by melittin, is a common process in eukaryotic cells, because unlike bacteria, they do not have a cell wall protecting their membranes from mechanical and chemical stress. Several repair mechanisms for membranes of eukaryotic cells and Entamoeba have been reported^67–69^. Throughout evolution eukaryotic cells have developed mechanisms to ensure their cell survival. For bacteria such repair mechanisms are reported less frequently because they are less susceptible due to their protective cell wall. Consistent with literature describing both transient and stable pore formation by melittin, depending on its concentration and orientation within the membrane, our data may also indicate that peptide penetration through the cell envelope is initially impaired. The observed increase in antimicrobial activity upon prolonged incubation suggests that access to the membrane, and thus the ability to locally accumulate melittin, is influenced by its retention time within the cell envelope. Furthermore, smaller pores or membrane damages (likely induced by insufficient local concentrations of melittin) can possibly be spontaneously resealed again, which could explain why, in our assays, membrane activity signals become fully saturated even at concentrations where cells retain full metabolic activity (Fig. 4). This supports the proposed mechanism of transient pore formation. As most bacteria are not known to have active repair mechanisms, stable toroidal pores probably cannot be resealed spontaneously anymore, leading to death of the bacterial cell^70^. Interestingly, surface charge neutralization along with outer membrane permeabilization suggests that binding to key anionic surface molecules, such as lipopolysaccharides (LPS), is central to melittin’s mode of action. As previously reported, melittin-damaged *E. coli* can survive in cytosol-mimicking environments^71^. Similarly, in our study, metabolic activity was still detectable at the LC_99.9_ concentration. These findings support that bacterial morphology can affect colony formation^72^. Indeed, colony-forming ability does not always reflect true viability, as metabolically active and membrane-intact cells may fail to form colonies under stress or membrane-compromising conditions, such as dormancy^73^.

The performed assays revealed for LL-37 a very sequential and clear mechanistic profile-it kills *E. coli* fast and independently of anionic phospholipids. Figure 7 shows that within the same time between 1.6 and 3.2 µM LL-37 is necessary to damage the both membranes and to kill the cells indicating that LL-37 does damage the membranes immediately and enough for actual cellular death. Interestingly, ATP content in the PG/CL depleted mutant had decreased markedly in the presence of low amount of LL-37, but remained constant even at higher concentrations. It is not unusual, that cells shut down their metabolism, remain arrested in a non-growing state and survive by a compensatory metabolism^74^. This is in contrast to programmed cell death-apoptosis in eucaryotes, which requires energy causing intracellular ATP level to increase dramatically^75^. In contrast, for LL-37 it was observed that ATP was depleted while the membrane permeabilization signals were almost saturated and the cells still exhibited prominent intracellular esterase activity. This gap in the timeline might indicate the ultimate target of LL-37 in the killing mechanism is beyond membrane damage, possibly involving intracellular components. Although its impact on ATP synthesis is not clear, but most likely by permeabilizing the bacterial membrane LL-37 may dissipate the proton motive force comprising the function of the ATP synthase^76^. It induces oxidative stress even before entering the cytosol^77^ and is highly active intracellularly, it disrupts cytoplasmic motion, and leads to collapse of cellular functions by electrostatically linking negatively charged DNA and ribosomes. This rigidification of the cytoplasm halts bacterial growth, which may help explain the slow development of resistance to AMPs^78^. AMPs acting on intracellular targets have been reported, for example Maculatin 1.1^79,80^. Most interestingly, LL-37 was unable to disrupt PE-rich membranes as demonstrate in Fig. 2 and by Sevcsik et al.^46^ raising question about its direct membrane-disruptive activity. Although it has been shown that LL-37 binds to LPS^81^ and the bacterial surface (Fig. 6), our results demonstrated that LL-37 did not completely neutralize the bacterial surface at LC_99.9_, indicating a threshold beyond which LL-37’s interaction with the bacterial surface does not further enhance its bactericidal effect. In fact, interactions with anionic phospholipids may be sufficient to destabilize the *E. coli* inner membrane by altering lipid packing and disrupting protein function and ion balance—similar to effects observed with other AMPs^31,32^.

In addition, our results suggest that—unless access to the outer and inner membranes is completely blocked (e.g., by a capsule or increased membrane density)—*E. coli* and similar bacteria have limited capacity to adapt their membrane composition in response to AMP attack due to their relatively simple lipid synthesis machinery^52^. This is exemplified by the restricted number of lipid classes detected in the BKT29 mutant, in which major anionic phospholipid synthesis is disrupted^53^. Under such stress conditions, membrane phospholipid synthesis shifts toward PA accumulation—a response commonly seen in both bacteria and eukaryotes. In yeast and mammals, blocking major phospholipid pathways leads to PA buildup^82^. Crucially, PA is a central lipid precursor and initiates triglyceride synthesis in eukaryotes. It constitutes one of the simplest subgroups of phospholipids and are found in cellular membranes in relatively small amount – approximately 1 mol % of total lipids. Regarding their biophysical properties, PA molecules have an inverted conical shape with a relatively small headgroup, which induces a negative spontaneous curvature^83^. No common PA-binding motif has been identified for interaction with various molecules (mainly proteins). However, short sequences containing lysine, arginine, and some hydrophobic residues have been shown to participate in PA binding. Interestingly, the interaction between the PA phosphate group and lysine or arginine residues increases the acidity of phosphate group, further enhancing the interaction^84^. This phenomenon has been widely described as an electrostatic/hydrogen bonding switch mechanism^85^. Given that melittin and LL-37 are also rich in basic and amphipathic residues, similar interactions with anionic lipids can be expected. Interestingly, our study confirms that both peptides do not merely associate with PA at the membrane surface but actively insert into PA-rich membranes, thereby supporting their pronounced membrane-permeabilizing activity observed in the BKT29 mutant. Additionally, melittin has been shown to induce inverse hexagonal phases in pure PA, further indicating its ability to destabilize membranes composed of or altered by the presence of PA^86,87^. In general, the enhanced activity of cationic peptides in the BKT29 mutant can be explained by complementary effects: PA maintains sufficient anionic character to attract the cationic peptide, while the absence of PG and CL may reduce surface sequestration, increasing local peptide availability and membrane disruption. In addition, loss of PG and CL likely compromises membrane robustness and overall cellular fitness, further sensitizing cells to peptide-induced killing. It should also be noted, while effects in whole cells may differ from model membranes due to proteins, glycans, and other cellular components, our study remains highly relevant, as the use of *E. coli* mutants lacking anionic phospholipids allowed direct assessment of how membrane composition influences AMP activity in a physiologically meaningful context. In conclusion, these findings suggest that AMPs LL-37 and melittin can effectively cope with lipid remodeling, reducing the likelihood of resistance arising from membrane composition changes.

In summary, the road to bacterial death induced by antimicrobial agents isn’t a one lane highway, where all events occur consecutively with clear separation of what happens first, but rather a big, complicated intersection where multiple actions happen in parallel, overlapping or even interfering with each other. In this context, the use of multiple assays is critically important for accurately describing the mode of action of AMPs as also emphasized by others^88^. All these actions associated with the cell’s viability could be reduced to the same denominator, yielding a comprehensible way of analyzing the mode of action of antimicrobial agents. Considering time, concentration and membrane composition antimicrobial profiles of membrane-active substances can be set up, describing the events leading to bacterial cell death by antimicrobial compounds correlating well with already published literature and therefore confirming reliable data with a broad interpretation and application potential. The alignment of the assays examined in this study and summarized build the fundament for future experiments, as the methods can be applied to other antimicrobial compounds and bacterial strains allowing a high-throughput examination of antimicrobials. Screening of antimicrobial activity of novel compounds can establish itself as a useful tool in development of new therapeutic strategies in the combat against multi-drug resistant infections.

## Methods

### Chemicals

The peptides melittin (GIGAVLKVLTTGLPALISWIKRKRQQ) and LL-37 (LLGDFFRKSKEKIGKEFKRIVQRIKDFLRNLVPRTES) were synthesized by PolyPeptide Group (USA). Melittin and LL-37 stock solutions were prepared in 0.1% acetic acid, pH 3.5, and the concentration of melittin additionally measured by NanoDrop™ UV-VIS Spectrophotometer (Peqlab Biotechnologie GmbH, Germany). 3,3′ -Dipropyl-thiadicarbocyanin iodide [DiSC_3_(5)] was purchased from Sigma-Aldrich (Austria) and propidium iodide (PI) solution in water from Invitrogen™ (Thermo Scientific, USA).

### Microorganisms

The following Gram-negative strains were used for all assays: *Escherichia coli* MG1655 (K-12) and its mutant strain BKT29^63,89,90^. BKT29 shows a depletion of the pgsA gene, clsABC, and ymdB genes. PgsA encodes a phosphatidylglycerol phosphate synthase, which is responsible for the catalysis of the PG synthesis from phosphatidic acid (PA). Both strains were a kind gift by Dr. Lorenzo Stella and Dr. Maria Luisa Mangoni (University of Roma Tor Vergata, Italy). All bacterial strains were stored in Mueller Hinton Bouillon (MHB) (Carl Roth, Germany) containing 20% (v/v) glycerol at -80°C.

### In silico interaction studies with membranes

To investigate how specific membrane lipids influence the interaction of LL-37 and melittin, we employed a local installation of AlphaFold 3^56^ to model peptide multimers in the presence of defined lipid compositions. Lipids were included via their CCD codes: PTY (phosphatidylethanolamine, PE, (1 R)-2-{[(S)-(2-aminoethoxy)(hydroxy)phosphoryl]oxy}-1-[(pentadecanoyloxy)methyl]ethyl icosanoate), P3A (phosphatidylglycerol, PG, [(2 R)-3-[[(2S)-2,3-dihydroxypropoxy]-oxido-phosphoryl]oxy-2-hydroxy-propyl] [(2 R)-3-hexadecanoyloxy-2-[(Z)-hexadec-9-enoyl]oxy-propyl] phosphate), CDL (cardiolipin, CL, [3-[[(2 ~ {R})-2,3-di(octadecanoyloxy)propoxy]-oxidanidyl-phosphoryl]oxy-2-oxidanyl-propyl] [(2 ~ {R})-2,3-di(octadecanoyloxy)propyl] phosphate), 3PH (phosphatic acid, PA, (2 R)-3-(phosphonooxy)propane-1,2-diyl dioctadecanoate), 4AG (DPPG, (2 R)-3-hydroxypropane-1,2-diyl dihexadecanoate) and PX8 (DSPG, (2 R)-2,3-bis(octadecanoyloxy)propyl hydrogen phosphate). For interaction with whole membranes, we used 14 molecules of the peptide with 100 molecules of lipids. In the case of individual interaction with a lipid we used one peptide molecule with one lipid molecule. Furthermore, peptide structures were predicted using AlphaFold 3 Version 4 on a local workstation equipped with an Nvidia GeForce RTX 3090 24 GB GPU, AMD Ryzen 9 5900X processor (12 cores), and 128 GB RAM, employing a deep learning-based architecture trained on experimentally determined structures to predict atomic-resolution models and provide per-residue confidence metrics (Beta Server, standard settings, 11 November 2024).

### Interaction of peptides with lipids by leakage assay and differential scanning calorimetry

Leakage assay was performed using liposomes composed of *E. coli* lipid extract, loaded with 8-aminonaphthalene-1,3,6-trisulfonic acid (ANTS) and p-xylene-bis-pyridinium bromide (DPX), as previously described^31,32^. The aqueous content released from the liposomes was monitored using an initial lipid concentration of 50 µM. Fluorescence emission was recorded over time before and after the addition of increasing concentrations of the peptide, ranging from 0.25 µM to 8 µM. Measurements were carried out using a VARIAN Cary Eclipse Fluorescence Spectrophotometer equipped with Cary Eclipse WinFLR Software (Agilent, Vienna, Austria). The percentage of leakage was calculated as the fluorescence intensity normalized to the maximum fluorescence obtained after the addition of 1% Triton X-100. Calorimetric experiments were performed in duplicates using a NanoDSC differential scanning calorimeter with a 0.3 mL capillary cell (TA instruments, New Castle, DE, USA) for pure 1,2-dipalmitoyl-sn-glycero-3-phosphate (PA) lipid, as well as for mixtures of PA with melittin and PA with LL-37. PA was obtained from Avanti (Sigma Aldrich, Austria). All measurements were conducted in filtered (0.22 µm) sodium phosphate-buffered saline (PBS) (20 mM Na_2_HPO_4_/NaH_2_PO_4_, 130 mM NaCl, pH 7.4). PA phospholipid was dissolved in a chloroform/methanol mixture (9:1 v/v) at a concentration of 10 mg/mL and aliquoted in smaller glass vials (100 µL each) to obtain 1 mg lipid films. The organic solvent was then evaporated under a gentle stream of nitrogen, and the resulting lipid films were incubated overnight under vacuum to remove any residual solvent. Next, the PA films were either dispersed in PBS buffer or in a melittin or LL-37 solution at 25:1 lipid-to-peptide molar ratio. The samples were hydrated at 75 °C (approximately 10 °C above the lipid phase transition temperature) for 1 hour with vigorous vortexing. All samples were left to rest at room temperature prior to measurement. Subsequently, six alternating heating and cooling scans were recorded for each sample, within a temperature range 10–80 °C and a scan rate 1 °C/min. Thermograms of the PBS buffer were subtracted from the excess heat capacity curves. Thermodynamic parameters were calculated and analyzed using Launch NanoAnalyze software v4.1.0 (TA Instruments, USA). The lipid melting temperature (T_m_) was defined as main transition temperature, and ΔH as main transition enthalpy. Two independent measurements were conducted using synthetic compounds of precisely known and stable concentration. Unlike biological samples, this minimized variability and resulted in low standard deviations between replicates, thereby eliminating the need for a third repetition.

### Cultivation of bacteria

Freshly grown cells were picked for overnight cultures (ONC), resuspended in MHB, and grown under shaking at 200 rpm and 37 °C. Optical density at 600 nm (OD_600_) was measured using a V-10 Plus Spectrophotometer (ONDA, Austria). Main cultures were inoculated at OD_600_ 0.05 and grown to mid-log phase for 3.5 h. The mid-log cells were then harvested by centrifugation (5 min, 3500 g) and washed two times using PBS. Determination of cell number was performed using the QUANTOM^™^ Total Cell Staining Kit (Biocat, Austria). The QUANTOM^™^ Total Cell Staining Dye penetrates through the membrane into the cell and gives a fluorescent signal upon binding to nucleic acids. It is able to stain live and dead cells, and thus provides information about the total cell number. The staining was performed according to protocol provided by the manufacturer. Briefly, the cell suspension was incubated in the dark with the included QUANTOM^™^ Total Cell Staining Dye and QUANTOM^™^ Total Cell Staining Enhancer for 1 min and applied to QUANTOM^™^ M50 Cell Counting Slide. Cells were counted by the QUANTOM™ Tx Microbial Cell Counter.

### General procedure for bacterial peptide treatment and assay preparation

Unless otherwise stated, all assays were performed in a similar manner. Washed mid-log bacterial cultures were diluted with PBS to a final concentration of 1 × 10^7^ cells mL^−1^. In 96-well microtiter plates 90 µl of the treated cells were then added to 10 µl peptide to yield a final concentration of 0.8–12.8 µM peptide. Water was used as a negative control, replacing peptide. As an additional control, peptides were tested without bacteria and showed no interference with dye fluorescence across the tested concentration range. For fluorescence-based assays, additional controls containing peptides without bacteria were included to confirm that the peptides did not interfere with dye signals. After treatment, cells were processed according to the specific assay protocol (e.g., PI uptake, ATP measurement, zeta potential, or metabolic activity).

### Antimicrobial activity

Assessment of antimicrobial activity was conducted by measuring bacterial growth in presence of the respective antimicrobial compound as a function of time. Washed mid-log *E. coli* cells were diluted in fresh MHB, which were then mixed with peptide and growth curves recorded for 24 h at 37 °C under shaking (300 rpm). All measurements were performed with the Bioscreen C (Oy Growth Curves Ab Ltd., Finland) at 420–580 nm. The lowest concentration of antimicrobial compound, where a 100% inhibition of bacterial growth could be observed, was defined as the IC_100%_ (inhibitory concentration). Alternatively, 1 × 10^7^ cells ml^−1^ mid-log growing cell were incubated with peptides for 1 or 2 h in PBS, before plated on MHB agar plates. Number of colonies was counted after 24 h at 37 °C and the lethal concentration LC_99.9_ estimated as the concentration of the peptides causing death in 99.9% of the initial bacterial population.

### Zeta potential

Mid-log phase cells were diluted to 1 × 10^7^ CFU/ml in HEPES buffer (10 mM HEPES, 140 mM NaCl, pH 7.4) and proceeded further similar to previously described protocols^20,21,30^. *E. coli* cells were treated with melittin and LL-37 for 5 min at room temperature and measured in a Zetasizer NANO (Malvern Instruments, Germany). Each measurement (*n* = 2) represents the average of 15 to 30 runs.

#### Membrane integrity: outer membrane permeability assay

The assay was performed in a similar fashion as previously described by Domadia et al.^91^ using 1-*N*-phenylnaphthylamine (NPN) as a marker. NPN is a non-polar probe, which fluoresces strongly in phospholipid environments, but not in aqueous environments. NPN is excluded by the outer membrane *of E. coli* unless it is severely compromised and is therefore used as an indicator for increased outer membrane permeability. The final concentration of NPN was 10 µM. The fluorescence reading was monitored by using a GloMax® Discover Microplate Reader (Promega, Austria) at an excitation wavelength of 365 nm and an emission wavelength range of 415–445 nm over 2 h at 37 °C. All measurements were performed in duplicates and all experiments repeated three times.

#### Membrane integrity: inner membrane permeability assay

The assay was adapted and modified from previously described experiments^92^, but was performed in analogy to our other assays. Propidium iodide (PI) was chosen as a marker as it is a nucleic acid intercalating agent, that can only penetrate damaged or severely compromised membranes^93^. The fluorescence signal of PI intensifies when binding to intracellular nucleic acids and is therefore used as an indicator for increased inner membrane permeability. Cells were treated with 2.5 µg ml^−1^ PI. The fluorescence reading was monitored by using a GloMax® Discover Microplate Reader (Promega, Austria) at an excitation wavelength of 520 nm and an emission wavelength range of 580–640 nm over 2 h at 37 °C. All measurements were performed in duplicates and all experiments repeated three times.

#### Membrane integrity: membrane depolarization assay

Changes in membrane potential of *E. coli* induced by membrane active compounds were assessed with the help of the cationic dye 3,3’ – Dipropylthiadicarbocyanine iodide (DiSC_3_ (5)). This hydrophobic and voltage sensitive dye embeds itself into the inner membrane, where the fluorescence is quenched and is dequenched when the membrane is depolarized, giving a fluorescent signal^58^. In comparison with the membrane permeability assay (PI Assay) the membrane depolarization assay is more sensitive to small changes in membrane integrity, which occur without massive disruption of the membrane. The assay was adapted from previously described experiments^94^. Mid-log *E. coli* cells were diluted in PBS containing 0.2 mM EDTA (pH 8.0) and 1 µM DiSC_3_(5) and then incubated for 30 min in the dark at room temperature. To equilibrate the cytoplasmic and external K^+^ concentration, 100 mM KCl was added after incubation. Prepared cells were further treated with peptides as described above. The fluorescence reading was monitored by using a Glomax Discovery fluorescence spectrophotometer at an excitation wavelength of 627 nm and an emission wavelength range of 660–720 nm over 2 h at 37 °C. All measurements were performed in duplicates and all experiments repeated three times.

#### Analysis of membrane integrity assays

Data evaluation was performed analogously to all Membrane Integrity Evaluation assays. For analysis of the experiments only data from a certain time interval, where signal values were constant, were taken into consideration. For this purpose all values, for every single concentration point, recorded between 30 and 60 min were averaged (I_t_) and the blank value (fluorescence intensity of cells without antimicrobial compound) subtracted (s. Equation 1). ∆I for every concentration point was then plotted against the concentration used of the respective antimicrobial compound, where I_t_ is averaged fluorescence intensity values for a specific time interval and I_0_ the fluorescence intensity for negative control (cells without antimicrobial compound).

1 $$\Delta {\rm{I}}={{\rm{I}}}_{{\rm{t}}}-{{\rm{I}}}_{0}$$

#### Cell viability assay based on intracellular esterase activity

For staining of live bacterial cells, the QUANTOM^™^ Viable Cell Staining Kit (Biocat, Germany) was used^22^. The QUANTOM™ Viable Cell Staining Dye provided in the kit is a Calcein AM derivative, which acts as a non-fluorescent esterase substrate. In the live cell, the substrate is promptly hydrolyzed by esterases to a fluorescent product. Therefore, intracellular esterase activity serves as an indicator of cell viability. Nine µl of 1 $\times$10^7 ^ml^−1^ *E. coli* mid-log cells were incubated for 1 h (37 °C, 300 rpm, Eppendorf T hermomixer C) with 1 µl peptide (melittin, LL-37) to a final concentration of 0.8–12.8 µM melittin/LL-37. After 1 h incubation live bacterial cells were counted according to protocol of the QUANTOM^™^ Viable Cell Staining Kit. Viable cell staining was performed by incubation with the QUANTOM^™^ Viable Cell Staining Dye for 10 min in the dark (37 °C, 300 rpm). Afterwards, live cells were counted by the QUANTOM™ Tx Microbial Cell Counter. The obtained cells/ml were then plotted against the antimicrobial compound concentration. All counts were performed in duplicate, and the entire experiment repeated three times.

#### Cell viability assay based on intracellular loss of metabolic activity (ATP)

To assess the metabolic activity of *E. coli* cells in presence of the respective membrane active compounds, the ATP content was determined by the BacTiter-Glo™ Microbial Cell Viability Assay (Promega, USA). The ATP present in the cell is here used as an indicator of metabolically active cells. It is extracted from bacterial cells by the BacTiter-Glo™ reagent, which at the same time contains also the Ultra-Glo™ Recombinant Luciferase and the substrate Luciferin needed for the reaction that eventually generates the luminescence signal. The assay relies on the light emission generated by the luciferase catalyzed mono-oxygenation of luciferin in the presence of ATP to oxyluciferin and AMP. Analogously to the previous experiments, the plates with treated bacteria were incubated for 1 h at 37 °C under shaking (200 rpm). After 1 h 100 µl of the BacTiter-Glo™ Reagent was added to all wells and the plates incubated for another 5 min. Luminescence was recorded by the GloMax® Discover Microplate Reader. The measured luminescence signal correlates directly with the ATP content. The ATP loss generated by treatment with the membrane active compounds was calculated using the equation below, where I_x_ is Luminescence intensity of cells treated with peptide, I_0_ is Luminescence intensity of cells treated with only water and I_buffer_ is Luminescence intensity of buffer.

2 $$\mathrm{ATP\; LOSS}\,[ \% ]=\frac{{{\rm{I}}}_{{\rm{x}}}-{{\rm{I}}}_{\mathrm{buffer}}}{{{\rm{I}}}_{0}-{{\rm{I}}}_{\mathrm{buffer}}}\cdot 100$$

For quantification of the ATP content an ATP standard curve was generated by preparing 10-fold serial dilutions ranging from 0.1 to 10^4^ nM of ATP and measuring luminescence according to protocol. The obtained values were plotted in a double logarithmic diagram and the regression line equation calculated. The ATP concentration present was calculated using Eq. 3, where [ATP] is calculated ATP concentration present [nM], I_x_ is Luminescence intensity of cells treated with X antimicrobial compound, I_0_ is Luminescence intensity of cells treated with only water, k is slope of calibration curve, d is intercept of calibration curve. The intercept d was subtracted from all measured intensities before calculating [ATP].

3 $$[\mathrm{ATP}]=\frac{{{\rm{I}}}_{{\rm{x}}}-{{\rm{I}}}_{0}}{{\rm{k}}}\cdot 100$$

Calibration curve was also performed with different bacterial concentrations (1 $\times$ 10^3^ –1 $\times$ 10^7^ cells ml^−1^, ten-fold steps) to obtain the limit of detection regarding bacterial cell numbers. The limit of detection was determined as approx. 1 $\times$ 10^5^ cells ml^−1^, as ATP content for bacterial concentrations lower than that could not be differentiated anymore from the background signal (buffer only). As an additional control, peptides were tested with assay conditions showing no interference with the luminescence across the tested concentration range.

#### Statistical analysis

Basal signal levels were measured in untreated bacterial cells or membrane models, serving as negative controls. To ensure reproducibility and comparability across conditions, all assays were normalized to a defined cell density prior to treatment. Most assays were conducted in duplicates within three independent experiments (*n* = 3), unless otherwise stated. Data are reported as mean ± standard deviation (SD), or median, as appropriate, unless otherwise stated. Drug effects were assessed across increasing concentrations, and conclusions were based on consistent, saturated signal responses. Welch’s t-test (performed in Excel) showed statistically significant differences (*p* < 0.005) between all peptide-treated conditions and the untreated control in membrane integrity and in esterase activity assays. However, for ATP measurements, reductions were observed in both wildtype and mutant strains, but statistical significance (*p* < 0.05) was only reached in the mutant strain at concentrations where the ATP signal stopped decreasing and plateaued. All calculations were performed using Microsoft Excel.

## Data Availability

All data are included in the manuscript. The original datasets generated during the current study are not publicly available because they are not deposited in a public repository but are available from the corresponding author upon reasonable request.

## References

[1] Devi, N. S. et al. Overview of antimicrobial resistance and mechanisms: The relative status of the past and current. *Microbe* **3**, 100083 (2024).

[2] Smith, W. P. J., Wucher, B. R., Nadell, C. D. & Foster, K. R. Bacterial defences: mechanisms, evolution and antimicrobial resistance. *Nat. Rev. Microbiol.* **21**, 519–534 (2023). 37095190 10.1038/s41579-023-00877-3

[3] Darby, E. M. et al. Molecular mechanisms of antibiotic resistance revisited. *Nat. Rev. Microbiol.* **21**, 280–295 (2023). 36411397 10.1038/s41579-022-00820-y

[4] Rosas, N. C. & Lithgow, T. Targeting bacterial outer-membrane remodelling to impact antimicrobial drug resistance. *Trends Microbiol.* **30**, 544–552 (2022). 34872824 10.1016/j.tim.2021.11.002

[5] Mansilla, M. C. & Mendoza, D. D. Regulation of Membrane Lipid Homeostasis in Bacteria upon Temperature Change. In *Biogenesis of Fatty Acids, Lipids and Membranes.* Handbook of Hydrocarbon and Lipid Microbiology. (eds Geiger, O.) pp.1–13, (Springer, Cham, 2016).

[6] Chwastek, G. et al. Principles of Membrane Adaptation Revealed through Environmentally Induced Bacterial Lipidome Remodeling. *Cell Rep.* **32**, 108165 (2020). 32966790 10.1016/j.celrep.2020.108165

[7] Agmon, E. & Stockwell, B. R. Lipid homeostasis and regulated cell death. *Curr. Opin. Chem. Biol.* **39**, 83–89 (2017). 28645028 10.1016/j.cbpa.2017.06.002 PMC5581689

[8] Winnikoff, J. R. et al. Homeocurvature adaptation of phospholipids to pressure in deep-sea invertebrates. *Science. (N. Y., N. Y.)* **384**, 1482–1488 (2024). 10.1126/science.adm7607 PMC11593575 38935710

[9] Wood, J. M. Bacterial responses to osmotic challenges. *J. Gen. Physiol.* **145**, 381–388 (2015). 25870209 10.1085/jgp.201411296 PMC4411257

[10] Malanovic, N. & Lohner, K. Antimicrobial Peptides Targeting Gram-Positive Bacteria. *Pharmaceuticals (Basel, Switzerland)* **9**, 59 (2016). 10.3390/ph9030059 PMC5039512 27657092

[11] Malanovic, N. & Lohner, K. Gram-positive bacterial cell envelopes: The impact on the activity of antimicrobial peptides. *Biochimica et. biophysica acta*. **1858**, 936–946 (2016). 26577273 10.1016/j.bbamem.2015.11.004

[12] Khondker, A. & Rheinstädter, M. C. How do bacterial membranes resist polymyxin antibiotics? *Commun. Biol.* **3**, 77 (2020). 32066819 10.1038/s42003-020-0803-x PMC7026071

[13] Global burden of bacterial antimicrobial resistance in 2019 a systematic analysis. *Lancet (Lond., Engl.)* **399**, 629–655 (2022). 10.1016/S0140-6736(21)02724-0 PMC8841637 35065702

[14] Hoenigl, M. et al. COVID-19-associated fungal infections. *Nat. Microbiol.* **7**, 1127–1140 (2022). 35918423 10.1038/s41564-022-01172-2 PMC9362108

[15] Shafran, N. et al. Secondary bacterial infection in COVID-19 patients is a stronger predictor for death compared to influenza patients. *Sci. Rep.* **11**, 12703 (2021). 34135459 10.1038/s41598-021-92220-0 PMC8209102

[16] Galeano Niño, J. L. et al. Effect of the intratumoral microbiota on spatial and cellular heterogeneity in cancer. *Nature* **611**, 810–817 (2022). 36385528 10.1038/s41586-022-05435-0 PMC9684076

[17] Malanovic, N. & Vejzovic, D. Novel insights at the crossroads of antibiotic use and cancer risk. *Cell stress* **7**, 46–49 (2023). 37265742 10.15698/cst2023.06.280 PMC10231269

[18] Narunsky-Haziza, L. et al. Pan-cancer analyses reveal cancer-type-specific fungal ecologies and bacteriome interactions. *Cell* **185**, 3789–3806.e17 (2022). 36179670 10.1016/j.cell.2022.09.005 PMC9567272

[19] Sinha, G. Tumors can teem with microbes. But what are they doing there? *Sci. (N. Y., N. Y.)* **378**, 693–694 (2022). 10.1126/science.adf8359 36395222

[20] Malanovic, N. et al. Disruption of the Cytoplasmic Membrane Structure and Barrier Function Underlies the Potent Antiseptic Activity of Octenidine in Gram-Positive Bacteria. *Appl. Environ. Microbiol.* **88**, 10.e0018022 (2022). 10.1128/aem.00180-22 PMC9128513 35481757

[21] Malanovic, N., Ön, A., Pabst, G., Zellner, A. & Lohner, K. Octenidine: Novel insights into the detailed killing mechanism of Gram-negative bacteria at a cellular and molecular level. *Int. J. antimicrobial agents* **56**, 106146 (2020). 10.1016/j.ijantimicag.2020.106146 32853670

[22] Vejzovic, D., Iftic, A., Ön, A., Semeraro, E. F. & Malanovic, N. Octenidine’s Efficacy: A Matter of Interpretation or the Influence of Experimental Setups? *Antibiotics (Basel, Switzerland)* **11**, 1665 (2022). 10.3390/antibiotics11111665 PMC9686575 36421309

[23] Dhoonmoon, L. & Malanovic, N. Enhancing patient outcomes: the role of octenidine-based irrigation solutions in managing sore and irritated peristomal skin. *J. wound care* **34**, S4–S11 (2025). 40314603 10.12968/jowc.2025.34.Sup4d.S4

[24] Bock, L. J. et al. Pseudomonas aeruginosa adapts to octenidine via a combination of efflux and membrane remodelling. *Commun. Biol.* **4**, 1058 (2021). 34504285 10.1038/s42003-021-02566-4 PMC8429429

[25] Lescat, M., Magnan, M., Kenmoe, S., Nordmann, P. & Poirel, L. Co-Lateral Effect of Octenidine, Chlorhexidine and Colistin Selective Pressures on Four Enterobacterial Species: A Comparative Genomic Analysis. *Antibiotics (Basel, Switzerland)* **11**, 50 (2021). 10.3390/antibiotics11010050 PMC8772718 35052927

[26] Dijksteel, G. S., Ulrich, M. M. W., Middelkoop, E. & Boekema, B. K. H. L. Review: Lessons Learned From Clinical Trials Using Antimicrobial Peptides (AMPs). *Front. Microbiol.* **12**, 616979 (2021). 33692766 10.3389/fmicb.2021.616979 PMC7937881

[27] Ridyard, K. E. & Overhage, J. The Potential of Human Peptide LL-37 as an Antimicrobial and Anti-Biofilm Agent. *Antibiotics (Basel, Switzerland)* **10**, 650 (2021). 10.3390/antibiotics10060650 PMC8227053 34072318

[28] de Breij, A. et al. The antimicrobial peptide SAAP-148 combats drug-resistant bacteria and biofilms. *Sci. Transl. Med.* **10**, 423 (2018). 10.1126/scitranslmed.aan4044 29321257

[29] Malanovic, N., Marx, L., Blondelle, S. E., Pabst, G. & Semeraro, E. F. Experimental concepts for linking the biological activities of antimicrobial peptides to their molecular modes of action. *Biochimica et. biophysica acta Biomembranes* **1862**, 183275 (2020). 32173291 10.1016/j.bbamem.2020.183275

[30] Piller, P. et al. Membrane Activity of LL-37 Derived Antimicrobial Peptides against Enterococcus hirae: Superiority of SAAP-148 over OP-145. *Biomolecules* **12**, 523 (2022). 10.3390/biom12040523 PMC9028586 35454112

[31] Ön, A. et al. Bactericidal Activity to Escherichia coli: Different Modes of Action of Two 24-Mer Peptides SAAP-148 and OP-145, Both Derived from Human Cathelicidine LL-37. *Antibiotics (Basel, Switzerland)* **12**, 1163 (2023). 10.3390/antibiotics12071163 PMC10376646 37508259

[32] Vejzovic, D. et al. Where Electrostatics Matter: Bacterial Surface Neutralization and Membrane Disruption by Antimicrobial Peptides SAAP-148 and OP-145. *Biomolecules* **12**, 1252 (2022). 10.3390/biom12091252 PMC9496175 36139091

[33] Wenzel, M. et al. Small cationic antimicrobial peptides delocalize peripheral membrane proteins. *Proc. Natl. Acad. Sci. USA* **111**, E1409–E1418 (2014). 24706874 10.1073/pnas.1319900111 PMC3986158

[34] Butler, M. S., Henderson, I. R., Capon, R. J. & Blaskovich, M. A. T. Antibiotics in the clinical pipeline as of December 2022. *J. Antibiotics* **76**, 431–473 (2023). 10.1038/s41429-023-00629-8 PMC10248350 37291465

[35] Zhang, H.-Q., Sun, C., Xu, N. & Liu, W. The current landscape of the antimicrobial peptide melittin and its therapeutic potential. *Front. Immunol.* **15**, 1326033 (2024). 38318188 10.3389/fimmu.2024.1326033 PMC10838977

[36] Kahlenberg, J. M. & Kaplan, M. J. Little peptide, big effects: the role of LL-37 in inflammation and autoimmune disease. *J. Immunol. (Baltim., Md.: 1950)* **191**, 4895–4901 (2013). 10.4049/jimmunol.1302005 PMC3836506 24185823

[37] Yang, L., Harroun, T. A., Weiss, T. M., Ding, L. & Huang, H. W. Barrel-stave model or toroidal model? A case study on melittin pores. *Biophysical J.* **81**, 1475–1485 (2001). 10.1016/S0006-3495(01)75802-X PMC1301626 11509361

[38] Oren, Z., Lerman, J. C., Gudmundsson, G. H., Agerberth, B. & Shai, Y. Structure and organization of the human antimicrobial peptide LL-37 in phospholipid membranes: relevance to the molecular basis for its non-cell-selective activity. *Biochemical J.* **341**, 501–513 (1999). PMC1220385 10417311

[39] Lee, C.-C., Sun, Y., Qian, S. & Huang, H. W. Transmembrane pores formed by human antimicrobial peptide LL-37. *Biophysical J.* **100**, 1688–1696 (2011). 10.1016/j.bpj.2011.02.018 PMC3072607 21463582

[40] Yang, Z., Choi, H. & Weisshaar, J. C. Melittin-Induced Permeabilization, Re-sealing, and Re-permeabilization of E. coli Membranes. *Biophysical J.* **114**, 368–379 (2018). 10.1016/j.bpj.2017.10.046 PMC5984949 29401434

[41] Sancho-Vaello, E. et al. The structure of the antimicrobial human cathelicidin LL-37 shows oligomerization and channel formation in the presence of membrane mimics. *Sci. Rep.* **10**, 17356 (2020). 33060695 10.1038/s41598-020-74401-5 PMC7562864

[42] Majewska, M., Zamlynny, V., Pieta, I. S., Nowakowski, R. & Pieta, P. Interaction of LL-37 human cathelicidin peptide with a model microbial-like lipid membrane. *Bioelectrochemistry (Amst., Neth.)* **141**, 107842 (2021). 10.1016/j.bioelechem.2021.107842 34049238

[43] Hong, J. et al. How Melittin Inserts into Cell Membrane: Conformational Changes, Inter-Peptide Cooperation, and Disturbance on the Membrane. *Molecules (Basel, Switzerland)* **24**, 1775 (2019). 10.3390/molecules24091775 PMC6539814 31067828

[44] Porcelli, F. et al. NMR structure of the cathelicidin-derived human antimicrobial peptide LL-37 in dodecylphosphocholine micelles. *Biochemistry* **47**, 5565–5572 (2008). 18439024 10.1021/bi702036s PMC5873590

[45] Sevcsik, E., Pabst, G., Jilek, A. & Lohner, K. How lipids influence the mode of action of membrane-active peptides. *Biochimica et. biophysica acta*. **1768**, 2586–2595 (2007). 17662236 10.1016/j.bbamem.2007.06.015

[46] Sevcsik, E. et al. Interaction of LL-37 with model membrane systems of different complexity: influence of the lipid matrix. *Biophysical J.* **94**, 4688–4699 (2008). 10.1529/biophysj.107.123620 PMC2397346 18326643

[47] van den Bogaart, G., Mika, J. T., Krasnikov, V. & Poolman, B. The lipid dependence of melittin action investigated by dual-color fluorescence burst analysis. *Biophysical J.* **93**, 154–163 (2007). 10.1529/biophysj.107.106005 PMC1914432 17434946

[48] Sochacki, K. A., Barns, K. J., Bucki, R. & Weisshaar, J. C. Real-time attack on single Escherichia coli cells by the human antimicrobial peptide LL-37. *Proc. Natl. Acad. Sci. USA* **108**, E77–E81 (2011). 21464330 10.1073/pnas.1101130108 PMC3080975

[49] White, J. K. et al. A stable cyclized antimicrobial peptide derived from LL-37 with host immunomodulatory effects and activity against uropathogens. *Cell. Mol. life Sci. : CMLS* **79**, 411 (2022). 35821354 10.1007/s00018-022-04440-w PMC9276586

[50] Strömstedt, A. A., Wessman, P., Ringstad, L., Edwards, K. & Malmsten, M. Effect of lipid headgroup composition on the interaction between melittin and lipid bilayers. *J. Colloid Interface Sci.* **311**, 59–69 (2007). 17383670 10.1016/j.jcis.2007.02.070

[51] Ulmschneider, J. P. & Ulmschneider, M. B. Melittin can permeabilize membranes via large transient pores. *Nat. Commun.* **15**, 7281 (2024). 39179607 10.1038/s41467-024-51691-1 PMC11343860

[52] Sohlenkamp, C. & Geiger, O. Bacterial membrane lipids: diversity in structures and pathways. *FEMS Microbiol. Rev.* **40**, 133–159 (2016). 25862689 10.1093/femsre/fuv008

[53] Savini, F. et al. Binding of an antimicrobial peptide to bacterial cells: Interaction with different species, strains and cellular components. *Biochimica et. biophysica acta Biomembranes* **1862**, 183291 (2020). 32234322 10.1016/j.bbamem.2020.183291

[54] Gunasekera, S., Muhammad, T., Strömstedt, A. A., Rosengren, K. J. & Göransson, U. Backbone Cyclization and Dimerization of LL-37-Derived Peptides Enhance Antimicrobial Activity and Proteolytic Stability. *Front. Microbiol.* **11**, 168 (2020). 32153522 10.3389/fmicb.2020.00168 PMC7046553

[55] Juhaniewicz-Debinska, J. Melittin-Induced Structural Transformations in DMPG and DMPS Lipid Membranes: A Langmuir Monolayer and AFM Study. *Molecules (Basel, Switzerland)* **29**, 6064 (2024). 10.3390/molecules29246064 PMC11677270 39770152

[56] Abramson, J. et al. Accurate structure prediction of biomolecular interactions with AlphaFold 3. *Nature* **630**, 493–500 (2024). 38718835 10.1038/s41586-024-07487-w PMC11168924

[57] Scheenstra, M. R. et al. Cathelicidins PMAP-36, LL-37 and CATH-2 are similar peptides with different modes of action. *Sci. Rep.* **9**, 4780 (2019). 30886247 10.1038/s41598-019-41246-6 PMC6423055

[58] Te Winkel, J. D., Gray, D. A., Seistrup, K. H., Hamoen, L. W. & Strahl, H. Analysis of Antimicrobial-Triggered Membrane Depolarization Using Voltage Sensitive Dyes. *Front. Cell Dev. Biol.* **4**, 29 (2016). 27148531 10.3389/fcell.2016.00029 PMC4829611

[59] Cantón, R. & Morosini, M.-I. Emergence and spread of antibiotic resistance following exposure to antibiotics. *FEMS Microbiol. Rev.* **35**, 977–991 (2011). 21722146 10.1111/j.1574-6976.2011.00295.x

[60] Kowalska-Krochmal, B. & Dudek-Wicher, R. The Minimum Inhibitory Concentration of Antibiotics: Methods, Interpretation, Clinical Relevance. *Pathogens (Basel, Switzerland)* **10**, 165 (2021). 10.3390/pathogens10020165 PMC7913839 33557078

[61] O’Neill, A. J. & Chopra, I. Preclinical evaluation of novel antibacterial agents by microbiological and molecular techniques. *Expert Opin. investigational drugs* **13**, 1045–1063 (2004). 10.1517/13543784.13.8.1045 15268641

[62] Loffredo, M. R. et al. Inoculum effect of antimicrobial peptides. *Proc. Natl. Acad. Sci. USA.* **118**, e2014364118 (2021). 10.1073/pnas.2014364118 PMC8166072 34021080

[63] Oliver, P. M. et al. Localization of anionic phospholipids in Escherichia coli cells. *J. Bacteriol.* **196**, 3386–3398 (2014). 25002539 10.1128/JB.01877-14 PMC4187673

[64] Pandidan, S. & Mechler, A. Nano-viscosimetry analysis of the membrane disrupting action of the bee venom peptide melittin. *Sci. Rep.* **9**, 10841 (2019). 31346251 10.1038/s41598-019-47325-y PMC6658469

[65] Wimley, W. C. How Does Melittin Permeabilize Membranes? *Biophysical J.* **114**, 251–253 (2018). 10.1016/j.bpj.2017.11.3738 PMC5984972 29401422

[66] Papo, N. & Shai, Y. Exploring peptide membrane interaction using surface plasmon resonance: differentiation between pore formation versus membrane disruption by lytic peptides. *Biochemistry* **42**, 458–466 (2003). 12525173 10.1021/bi0267846

[67] Ramírez-Montiel, F. et al. Plasma membrane damage repair is mediated by an acid sphingomyelinase in Entamoeba histolytica. *PLoS Pathog.* **15**, e1008016 (2019). 31461501 10.1371/journal.ppat.1008016 PMC6713333

[68] Blazek, A. D., Paleo, B. J. & Weisleder, N. Plasma Membrane Repair: A Central Process for Maintaining Cellular Homeostasis. *Physiol. (Bethesda, Md.)* **30**, 438–448 (2015). 10.1152/physiol.00019.2015 PMC4630197 26525343

[69] Daussy, C. F. & Wodrich, H. “Repair Me if You Can”: Membrane Damage, Response, and Control from the Viral Perspective. *Cells* **9,** 2042 (2020). 10.3390/cells9092042 PMC7564661 32906744

[70] Lee, M.-T., Sun, T.-L., Hung, W.-C. & Huang, H. W. Process of inducing pores in membranes by melittin. *Proc. Natl. Acad. Sci. USA* **110**, 14243–14248 (2013). 23940362 10.1073/pnas.1307010110 PMC3761581

[71] Kamiura, R., Matsuda, F. & Ichihashi, N. Survival of membrane-damaged Escherichia coli in a cytosol-mimicking solution. *J. Biosci. Bioeng.* **128**, 558–563 (2019). 31182278 10.1016/j.jbiosc.2019.05.005

[72] Sreepadmanabh, M. et al. Cell shape affects bacterial colony growth under physical confinement. *Nat. Commun.* **15**, 9561 (2024). 39516204 10.1038/s41467-024-53989-6 PMC11549454

[73] Trinh, K. T. L. & Lee, N. Y. Recent Methods for the Viability Assessment of Bacterial Pathogens: Advances, Challenges, and Future Perspectives. *Pathogens (Basel, Switzerland)* **11**, 1057 (2022). 10.3390/pathogens11091057 PMC9500772 36145489

[74] Lempp, M., Lubrano, P., Bange, G. & Link, H. Metabolism of non-growing bacteria. *Biol. Chem.* **401**, 1479–1485 (2020). 32845858 10.1515/hsz-2020-0201

[75] Zamaraeva, M. V. et al. Cells die with increased cytosolic ATP during apoptosis: a bioluminescence study with intracellular luciferase. *Cell Death Differ.* **12**, 1390–1397 (2005). 15905877 10.1038/sj.cdd.4401661

[76] Farha, M. A., Verschoor, C. P., Bowdish, D. & Brown, E. D. Collapsing the proton motive force to identify synergistic combinations against Staphylococcus aureus. *Chem. Biol.* **20**, 1168–1178 (2013). 23972939 10.1016/j.chembiol.2013.07.006

[77] Choi, H., Yang, Z. & Weisshaar, J. C. Oxidative stress induced in E. coli by the human antimicrobial peptide LL-37. *PLoS Pathog.* **13**, e1006481 (2017). 28665988 10.1371/journal.ppat.1006481 PMC5509375

[78] Zhu, Y., Mohapatra, S. & Weisshaar, J. C. Rigidification of the Escherichia coli cytoplasm by the human antimicrobial peptide LL-37 revealed by superresolution fluorescence microscopy. *Proc. Natl. Acad. Sci. USA* **116**, 1017–1026 (2019). 30598442 10.1073/pnas.1814924116 PMC6338858

[79] Sani, M.-A., Henriques, S. T., Weber, D. & Separovic, F. Bacteria May Cope Differently from Similar Membrane Damage Caused by the Australian Tree Frog Antimicrobial Peptide Maculatin 1.1. *J. Biol. Chem.* **290**, 19853–19862 (2015). 26100634 10.1074/jbc.M115.643262 PMC4528145

[80] Separovic, F., Hofferek, V., Duff, A. P., McConville, M. J. & Sani, M.-A. In-cell DNP NMR reveals multiple targeting effect of antimicrobial peptide. *J. Struct. Biol.: X* **6**, 100074 (2022). 36147732 10.1016/j.yjsbx.2022.100074 PMC9486116

[81] Nell, M. J. et al. Development of novel LL-37 derived antimicrobial peptides with LPS and LTA neutralizing and antimicrobial activities for therapeutic application. *Peptides* **27**, 649–660 (2006). 16274847 10.1016/j.peptides.2005.09.016

[82] Malanovic, N. et al. S-adenosyl-L-homocysteine hydrolase, key enzyme of methylation metabolism, regulates phosphatidylcholine synthesis and triacylglycerol homeostasis in yeast: implications for homocysteine as a risk factor of atherosclerosis. *J. Biol. Chem.* **283**, 23989–23999 (2008). 18591246 10.1074/jbc.M800830200 PMC3259781

[83] Zegarlińska, J., Piaścik, M., Sikorski, A. F. & Czogalla, A. Phosphatidic acid - a simple phospholipid with multiple faces. *Acta biochimica Polonica* **65**, 163–171 (2018). 29913482 10.18388/abp.2018_2592

[84] Kulig, W. et al. Complex Behavior of Phosphatidylcholine-Phosphatidic Acid Bilayers and Monolayers: Effect of Acyl Chain Unsaturation. *Langmuir: ACS J. Surf. colloids* **35**, 5944–5956 (2019). 10.1021/acs.langmuir.9b00381 30942590

[85] Eaton, J. M. et al. Lipin 2 binds phosphatidic acid by the electrostatic hydrogen bond switch mechanism independent of phosphorylation. *J. Biol. Chem.* **289**, 18055–18066 (2014). 24811178 10.1074/jbc.M114.547604 PMC4140300

[86] Bechinger, B. The structure, dynamics and orientation of antimicrobial peptides in membranes by multidimensional solid-state NMR spectroscopy. *Biochimica et. biophysica acta*. **1462**, 157–183 (1999). 10590307 10.1016/s0005-2736(99)00205-9

[87] Batenburg, A. M. et al. Interaction of melittin with negatively charged phospholipids: consequences for lipid organization. *FEBS Lett.* **223**, 148–154 (1987). 3666135 10.1016/0014-5793(87)80526-4

[88] Wang, X. et al. Analyzing mechanisms of action of antimicrobial peptides on bacterial membranes requires multiple complimentary assays and different bacterial strains. *Biochimica et. biophysica acta Biomembranes*. **1865**, 184160 (2023). 37100361 10.1016/j.bbamem.2023.184160

[89] Agrawal, A., Rangarajan, N. & Weisshaar, J. C. Resistance of early stationary phase E. coli to membrane permeabilization by the antimicrobial peptide Cecropin A. *Biochimica et. biophysica acta Biomembranes* **1861**, 182990 (2019). 31129116 10.1016/j.bbamem.2019.05.012 PMC6721983

[90] Tan, B. K. et al. Discovery of a cardiolipin synthase utilizing phosphatidylethanolamine and phosphatidylglycerol as substrates. *Proc. Natl. Acad. Sci. USA* **109**, 16504–16509 (2012). 22988102 10.1073/pnas.1212797109 PMC3478633

[91] Domadia, P. N., Bhunia, A., Ramamoorthy, A. & Bhattacharjya, S. Structure, interactions, and antibacterial activities of MSI-594 derived mutant peptide MSI-594F5A in lipopolysaccharide micelles: role of the helical hairpin conformation in outer-membrane permeabilization. *J. Am. Chem. Soc.* **132**, 18417–18428 (2010). 21128620 10.1021/ja1083255

[92] Chitolie, M. S. & Toescu, E. C. High-throughput method for dynamic measurements of cellular viability using a BMG LABTECH microplate reader. *The BMG LABTECH* (2008).

[93] Crowley, L. C. et al. Measuring Cell Death by Propidium Iodide Uptake and Flow Cytometry. *Cold Spring Harb. protoc.* **2016**, 10.1101/pdb.prot087163 (2016). 10.1101/pdb.prot087163 27371595

[94] Scheinpflug, K. et al. Antimicrobial peptide cWFW kills by combining lipid phase separation with autolysis. *Sci. Rep.* **7**, 44332 (2017). 28276520 10.1038/srep44332 PMC5343580

